# Community-Based First Responder Network in Rural Thailand: A Case Study of Out-of-Hospital Cardiac Arrest

**DOI:** 10.1017/S1049023X20001545

**Published:** 2021-04

**Authors:** Ratrawee Pattanarattanamolee, Rujeewan Yomstreeken Sanglun, Shinji Nakahara

**Affiliations:** 1. Khon Kaen Regional Hospital, Khon Kaen, Thailand; 2. Khon Kaen Provincial Health Office, Khon Kaen, Thailand; 3. Graduate School of Health Innovation, Kanagawa University of Human Services, Kawasaki, Japan

**Keywords:** first aid, lay volunteer, low- and middle-income countries, prehospital care, primary health care

## Abstract

Recently, the Thai government introduced a novel program to train health volunteers as first responders to deal with increasing acute illness and injuries. This case study demonstrates the potential of this program to improve public access to emergency care through the integration of emergency care with a community-based health care system, specifically in a rural setting. A 39-year-old man collapsed with cardiac arrest in his village. Lay first responders from his neighborhood attended him immediately, administered chest compressions, and contacted Emergency Medical Services (EMS). They continued chest compressions until the EMS unit arrived. While the EMS transported him to the hospital, the patient attained return of spontaneous circulation and consciousness. He returned to his normal life without obvious neurological problems. The Thai strategy to develop a community-based first responder network through health volunteer training would address the issue of inequitable access to emergency care and improve patients’ chances of survival and prognoses.

## Introduction

Timely chest compression can considerably increase the possibility of neurologically intact survival of out-of-hospital cardiac arrest (OHCA) patients.^1^ Basic resuscitation training to the general public has been widely implemented to encourage bystander chest compression in events of cardiac arrest. However, first responder training to selected groups of motivated volunteers is promising; certain occupational groups with a high probability of encountering severely injured or ill patients have a motivation to help such patients.^2^ The first responders can complement the role of the general public, particularly where the possibility of resuscitation by the general public is low; this could be due to a small proportion of trained population or high perceived barriers to performing chest compression, such as fear of harming the individual or litigation if compressions fail.^3^

Recently, the Thai Emergency Medical Services (EMS) system has emphasized organizing first responders as a community-level EMS component in addition to increasing municipality-based EMS units that employ basic-level personnel and equipment.^4^ The first responder training has been provided to health volunteers, police officers, firefighters, teachers, and community leaders; collectively, they are called community emergency volunteers (CEVs).^5^ Their primary role is to minimize inequitable access to emergency care, particularly in resource-constrained areas.

This specialized training to health volunteers, who are providing basic health care to their communities, is a novel program that integrates emergency care with a community-based health care system. However, this program has only been briefly described;^5^ there is a lack of studies that have evaluated its potential and effectiveness.

This case study presents a successful example of lay first responders’ prompt aid to an OHCA patient, depicting the effectiveness of the training program. Further discussed are the possible applications of this strategy to other resource-constrained settings.

## Case Presentation

This case occurred in a rural village in north-eastern Thailand in June 2016. A 39-year-old man with no known history of serious health problems became unconscious and collapsed in front of his house. A witness shouted for help, and a village health volunteer (VHV), in charge of basic health care in that village and within the earshot of the witness, immediately attended the patient. She had received a one-day training for emergency first aid to be a CEV. She rapidly assessed his condition, noticed his deep coma and lack of respiration or pulse, and immediately administered chest compressions. Another CEV, also responding to the shout, called EMS. The two CEVs continued chest compression in turn for six minutes until an ambulance arrived.

Based on the information from the CEV, the dispatcher in the command control center of the provincial EMS system activated a dual response. The nearest municipality-based EMS unit was dispatched from the sub-district office, and an advanced-level EMS unit was dispatched from the nearest district hospital. The municipality-based unit arrived at the scene first.

The EMS personnel confirmed the patient’s cardiac arrest and administered chest compressions and ventilation using a bag-valve-mask. They transported him to the district hospital with a plan to meet the advanced-level team on the way. Several minutes later, the patient attained return of spontaneous circulation and regained consciousness before the rendezvous with the advanced-level team. Because the patient fully recovered his heartbeat and consciousness and he strongly refused to go to the hospital, the ambulance returned to the village. The patient then returned to his everyday life without any obvious health problems, as observed by the VHV in the days following the incident. Written informed consent was obtained from the patient for this case study.

## Discussion

The successful survival of the present case indicates the potential of mobilizing existing community-based health care resources to improve access to emergency care in remote areas. The trained CEVs in the patient’s neighborhood immediately initiated chest compressions and contacted EMS, constituting the important components of the chain of survival.^1^ In addition, the CEVs are ubiquitous in the communities and can minimize inequitable access to emergency care, considering the long EMS response time in many rural areas.

The CEVs in this case study also served as VHVs who are the most peripheral health care personnel. They provide outreach services to people as the first points of contact to the health care system. The VHVs are community residents who receive a seven-day training and are responsible for approximately ten households in their neighborhood.^6^ Their health care services include providing health education and information, connecting those with health problems to health care facilities, and collecting health data. The community people can contact them regarding their health problems.

Many low- and middle-income countries (LMICs) with insufficient health care resources have adopted community-based strategies known as primary health care to expand their health care coverage. Instead of investing in hospital-based care and formally trained staff, they trained lay people in the community as community-based health care personnel and delegated many health care tasks to them.^7^ These non-professional health care providers are known as community health workers or volunteers. Thailand is one of the successful countries in improving public access to health care following this strategy with its excellent network of VHVs.^6^

Formerly, the community-based strategies focused on maternal and child health care and infection control; this has now expanded to non-communicable diseases and injuries following epidemiological transitions in LMICs.^8^ Integrating emergency care components into general health services has proven to be efficient in strengthening the existing health care system.^9^

The National Institute of Emergency Medicine (NIEM; Nonthaburi, Thailand), the national agency for emergency care, started the CEV program to establish a community-based emergency care network. This program aims to train one percent of the population as CEVs, who serve as the first points of contact to emergency care.^4,10^ These CEVs are selected from VHVs, police officers, firefighters, teachers, and community leaders.^5^ The CEV training curriculum includes: (1) how to call the EMS system using the nation-wide unique four-digit phone number of 1669; (2) how to assess and manage serious conditions, such as stroke, myocardial infarction, cardiac arrest, and trauma; (3) judging when to call EMS; and (4) how to provide first aid, for example, wound dressing and splinting to trauma patients and chest compression to cardiac arrest patients.^5^

These CEVs, despite their informal nature, constitute the basic community-level prehospital care system and considerably improve access to emergency care, particularly in remote areas.^4^ If VHVs in every neighborhood are trained as CEVs, they can promptly attend the patient and provide timely first aid while waiting for the formal EMS team. Many CEVs are also VHVs; improving their skills through emergency care training would increase their trustworthiness in the villages.

The lay first responders can supplement the training programs for the general public.^2^ The proportion of those who can provide basic resuscitation is still extremely low, particularly in rural areas in Thailand, despite the NIEM-implemented first aid training courses for the general public at schools and workplaces. While it will take time to increase the number of people who receive first aid training among the general public, first responder programs can efficiently provide care to those who need it. Furthermore, first responders are highly motivated volunteers who would feel lesser barriers than the general public, which sometimes deter them from helping sick patients.

In a resource-constrained setting, integrating first aid training into existing community-based health care programs is a promising and efficient strategy. Community health workers are already providing general health services and are highly motivated to overcome perceived barriers. Additionally, the training is not costly. The first responders’ intervention can provide timely assistance while waiting for the EMS to arrive from a distant place.

## Conclusions

An OHCA case in a rural Thai village survived after receiving timely and life-saving chest compressions by two first responders, trained as VHVs/CEVs. Thai strategies to develop community-based first responder networks through volunteer training programs have the potential to address inequitable access to emergency care, particularly in areas with poor access to formal EMS and hospitals.
